# Supplementary material for: Higher momentary parental burnout predicts lower subsequent emotional expression in parents during the festive season
Source: Commun Psychol. 2025 Nov 24;3:167. doi: 10.1038/s44271-025-00346-y (PMC12644506; doi:10.1038/s44271-025-00346-y)
Supplement: Supplementary file 2 — Supplementary Equation [file 44271_2025_346_MOESM2_ESM.pdf]

Supplementary Equation 1

Decomposition

$$\begin{cases} PB_{ti} = PB_{ti}^{(w)} + PB_{i}^{(b)} \\ GE_{ti} = GE_{ti}^{(w)} + GE_{i}^{(b)} \end{cases}$$

Within-Person

$$\begin{cases} PB_{ti}^{(w)} = \varphi_{1i} \times PB_{t-1,i}^{(w)} + \varphi_{3i} \times GE_{t-1,i}^{(w)} + e_{1ti} \\ GE_{ti}^{(w)} = \varphi_{2i} \times GE_{t-1,i}^{(w)} + \varphi_{4i} \times PB_{t-1,i}^{(w)} + e_{2ti} \\ e_{1ti} \sim N\left(0, \sigma_{PB,i}^{2}\right) \\ e_{2ti} \sim N\left(0, \sigma_{GE,i}^{2}\right) \end{cases}$$

Between-Person

$$\begin{cases}
PB_{i}^{(b)} = \gamma_{00} + a_1 \times PB_{i}^{(Baseline)} + \\
\qquad \gamma_{01} \times Mother_i + \gamma_{02} \times Needs_i + \gamma_{03} \times Single_i + \gamma_{04} \times Income_i + \zeta_{0i} \\
\ln\left(\sigma_{PB,i}^{2}\right) = \gamma_{10} + a_2 \times PB_{i}^{(Baseline)} + \\
\qquad \gamma_{11} \times Mother_i + \gamma_{12} \times Needs_i + \gamma_{13} \times Single_i + \gamma_{14} \times Income_i + \zeta_{1i} \\
\varphi_{1i} = \gamma_{20} + a_3 \times PB_{i}^{(Baseline)} + \\
\qquad \gamma_{21} \times Mother_i + \gamma_{22} \times Needs_i + \gamma_{23} \times Single_i + \gamma_{24} \times Income_i + \zeta_{2i} \\
\varphi_{2i} = \gamma_{30} + a_4 \times GE_{i}^{(Baseline)} + \\
\qquad \gamma_{31} \times Mother_i + \gamma_{32} \times Needs_i + \gamma_{33} \times Single_i + \gamma_{34} \times Income_i + \zeta_{3i} \\
\varphi_{3i} = \gamma_{40} + \zeta_{4i} \\
\varphi_{4i} = \gamma_{50} + \zeta_{5i} \\
GE_{i}^{(b)} = \gamma_{60} + a_5 \times GE_{i}^{(Baseline)} + \\
\qquad \gamma_{61} \times Mother_i + \gamma_{62} \times Needs_i + \gamma_{63} \times Single_i + \gamma_{64} \times Income_i + \zeta_{6i} \\
\ln\left(\sigma_{GE,i}^{2}\right) = \gamma_{70} + a_6 \times GE_{i}^{(Baseline)} + \\
\qquad \gamma_{71} \times Mother_i + \gamma_{72} \times Needs_i + \gamma_{73} \times Single_i + \gamma_{74} \times Income_i + \zeta_{7i} \\
PB_{i}^{(Follow)} = \gamma_{80} + b_1 \times PB_{i}^{(b)} + b_2 \times \ln\left(\sigma_{PB,i}^{2}\right) + b_3 \times \varphi_{1i} + c'_1 \times PB_{i}^{(Baseline)} + \beta_1 \times GE_{i}^{(Baseline)} + \\
\qquad \gamma_{81} \times Mother_i + \gamma_{82} \times Needs_i + \gamma_{83} \times Single_i + \gamma_{84} \times Income_i + \zeta_{8i} \\
GE_{i}^{(Follow)} = \gamma_{90} + b_4 \times \varphi_{2i} + b_5 \times GE_{i}^{(b)} + b_6 \times \ln\left(\sigma_{GE,i}^{2}\right) + c'_2 \times GE_{i}^{(Baseline)} + \beta_2 \times PB_{i}^{(Baseline)} + \\
\qquad \gamma_{91} \times Mother_i + \gamma_{92} \times Needs_i + \gamma_{93} \times Single_i + \gamma_{94} \times Income_i + \zeta_{9i} \\
PB_{i}^{(Baseline)} = \gamma_{10,0} + \\
\qquad \gamma_{10,1} \times Mother_i + \gamma_{10,2} \times Needs_i + \gamma_{10,3} \times Single_i + \gamma_{10,4} \times Income_i + \zeta_{10i} \\
GE_{i}^{(Baseline)} = \gamma_{11,0} + \\
\qquad \gamma_{11,1} \times Mother_i + \gamma_{11,2} \times Needs_i + \gamma_{11,3} \times Single_i + \gamma_{11,4} \times Income_i + \zeta_{11i} \\
\boldsymbol{\zeta}_i \sim MVN\left(\mathbf{0}_{12}, \begin{bmatrix}
\psi_{00} \\
\psi_{10} & \psi_{11} \\
0 & 0 & \psi_{22} \\
\vdots & \ddots & 0 & \psi_{33} \\
\vdots & & \ddots & 0 & \psi_{44} \\
\vdots & & & \ddots & 0 & \psi_{55} \\
\vdots & & & & \ddots & 0 & \psi_{66} \\
\vdots & & & & & \ddots & \psi_{76} & \psi_{77} \\
\vdots & & & & & & \ddots & 0 & \psi_{88} \\
\vdots & & & & & & & \ddots & \psi_{98} & \psi_{99} \\
\vdots & & & & & & & & \ddots & 0 & \psi_{10,10} \\
0 & \dots & \dots & \dots & \dots & \dots & \dots & \dots & \dots & 0 & \psi_{11,10} & \psi_{11,11}
\end{bmatrix}\right)
\end{cases}$$
