# Supplementary material for: Higher momentary parental burnout predicts lower subsequent emotional expression in parents during the festive season
Source: Commun Psychol. 2025 Nov 24;3:167. doi: 10.1038/s44271-025-00346-y (PMC12644506; doi:10.1038/s44271-025-00346-y)
Supplement: Supplementary file 3 — Reporting Summary [file 44271_2025_346_MOESM3_ESM.pdf]

nature portfolio

Corresponding author(s): Ziwen Teuber

Last updated by author(s): Sep 6, 2025

Reporting Summary

Nature Portfolio wishes to improve the reproducibility of the work that we publish. This form provides structure for consistency and transparency in reporting. For further information on Nature Portfolio policies, see our Editorial Policies and the Editorial Policy Checklist.

Statistics

For all statistical analyses, confirm that the following items are present in the figure legend, table legend, main text, or Methods section.

n/a	Confirmed	
☐	☒	The exact sample size (n) for each experimental group/condition, given as a discrete number and unit of measurement
☐	☒	A statement on whether measurements were taken from distinct samples or whether the same sample was measured repeatedly
☒	☐	The statistical test(s) used AND whether they are one- or two-sided<br>*Only common tests should be described solely by name; describe more complex techniques in the Methods section.*
☐	☒	A description of all covariates tested
☐	☒	A description of any assumptions or corrections, such as tests of normality and adjustment for multiple comparisons
☐	☒	A full description of the statistical parameters including central tendency (e.g. means) or other basic estimates (e.g. regression coefficient) AND variation (e.g. standard deviation) or associated estimates of uncertainty (e.g. confidence intervals)
☐	☒	For null hypothesis testing, the test statistic (e.g. F, t, r) with confidence intervals, effect sizes, degrees of freedom and P value noted<br>*Give P values as exact values whenever suitable.*
☐	☒	For Bayesian analysis, information on the choice of priors and Markov chain Monte Carlo settings
☐	☒	For hierarchical and complex designs, identification of the appropriate level for tests and full reporting of outcomes
☐	☒	Estimates of effect sizes (e.g. Cohen's d, Pearson's r), indicating how they were calculated

Our web collection on statistics for biologists contains articles on many of the points above.

Software and code

Policy information about availability of computer code

Data collection	Participants were recruited via the Prolific platform. To receive study prompts, they downloaded the Mindsampler application, within which Qualtrics surveys were embedded.
Data analysis	We used R to prepare data and Mplus to analyze data.

For manuscripts utilizing custom algorithms or software that are central to the research but not yet described in published literature, software must be made available to editors and reviewers. We strongly encourage code deposition in a community repository (e.g. GitHub). See the Nature Portfolio guidelines for submitting code & software for further information.

Data

Policy information about availability of data

All manuscripts must include a data availability statement. This statement should provide the following information, where applicable:

- Accession codes, unique identifiers, or web links for publicly available datasets
- A description of any restrictions on data availability
- For clinical datasets or third party data, please ensure that the statement adheres to our policy

Data and analysis codes associated with this study are available at https://osf.io/5dnrm/?view_only=dd92d3260d264eb08251c3af32678a92.

Research involving human participants, their data, or biological material

Policy information about studies with human participants or human data. See also policy information about sex, gender (identity/presentation), and sexual orientation and race, ethnicity and racism.

Reporting on sex and gender	Participants were asked to indicate their gender (male, female, genderfluid/nonbinary/agender, or do not wish to say). Of the respondents, 188 identified as female and 105 as male."
Reporting on race, ethnicity, or other socially relevant groupings	24 participants (8.11%) identified as single parents, while the remaining 269 were married or in a committed relationship. A total of 62 parents (21.16%) reported having a child with special educational needs (e.g., ADHD, learning disabilities).<br>Data on race/ethnicity was not collected.
Population characteristics	Participants were parents from the United Kingdom, each with at least one child under the age of 10. Their average age was 38.16 years (SD = 6.92). The sample comprised 188 mothers and 105 fathers. Additionally, 24 participants (8.11%) identified as single parents, while the remaining 269 were married or in a committed relationship. The median annual household net income fell within the range of £50,000–£59,999, which was higher than the UK's median household income of £34,500 in 2023 (Office for National Statistics, 2024). On average, households had 1.86 children (SD = 0.83). A total of 62 parents (21.16%) reported having a child with special educational needs (e.g., ADHD, learning disabilities).
Recruitment	The study was conducted from November 2023 to January 2024 and comprised a baseline survey, a 35-day experience sampling period, and a follow-up survey, respectively conducted before, during, and after the 2023 Christmas festive season. In November 2023, 380 parents from the United Kingdom, each with at least one child under the age of 10, were recruited via the cloud-sourcing platform Prolific. They first completed a baseline survey two weeks before being invited to download the MindSampler app on November 30, 2023. This application is designed for collecting intensive longitudinal data and integrates Qualtrics surveys. Participants received three randomly timed daily prompts between 8:00 and 20:00, from November 30, 2023 (Day 1) to January 3, 2024 (Day 35). Of those invited, 315 participants completed at least 30 daily experience sampling surveys throughout the study. On January 15, 2024, 307 of these participants also completed the follow-up survey. Participants received a total compensation of £30, distributed as follows: £4 for completing the baseline survey, £1 for installing the MindSampler app, £10 as a flat participation fee for responding to at least 30 notifications during the 35-day experience sampling period, and £5 for completing the follow-up survey. Additionally, participants who completed all three parts of the study received a £10 bonus. The intensive longitudinal design required high participant engagement, which may have resulted in a sample of parents with better self-regulatory skills than the average population. This introduces potential selection bias, as parents with lower self-regulation or coping skills might be less likely to participate.
Ethics oversight	Ethics Review Committee of the University of Vienna; protocol number: 2023/W/021

Note that full information on the approval of the study protocol must also be provided in the manuscript.

Field-specific reporting

Please select the one below that is the best fit for your research. If you are not sure, read the appropriate sections before making your selection.

☐ Life sciences ☒ Behavioural & social sciences ☐ Ecological, evolutionary & environmental sciences

For a reference copy of the document with all sections, see nature.com/documents/nr-reporting-summary-flat.pdf

Behavioural & social sciences study design

All studies must disclose on these points even when the disclosure is negative.

Study description	Intensive longitudinal study (experience sampling) with pre- and post-surveys
Research sample	The sample consisted of 293 U.K. parent from distinct family units (i.e., no parent dyads), with an average age of 38.16 years (SD = 6.92). The sample comprised 188 mothers and 105 fathers. Additionally, 24 participants (8.11%) identified as single parents, while the remaining 269 were married or in a committed relationship. The median annual household net income fell within the range of £50,000–£59,999, which was higher than the UK's median household income of £34,500 in 2023 (Office for National Statistics, 2024). On average, households had 1.86 children (SD = 0.83). A total of 62 parents (21.16%) reported having a child with special educational needs (e.g., ADHD, learning disabilities).
Sampling strategy	We recruited participants via Prolific. We invited 380 participants, who were eligible, to complete the presurvey. Regarding sample size, as stated in the preregistration, we did not conduct an a priori power analysis because our recruitment strategy aimed to include as many parents as possible within the available resources. Furthermore, Sultzberg and Muthén (2018) conducted power simulations for common designs to suggest general guidelines for adequate sample sizes. The focal effects in our study correspond to between-person predictors of a residual variance or residual variance as a mediator. Accordingly, for 50 time points per person, adequate power to detect between-person effects on a log-residual variance is achieved with N = 100, well below our sample size of 293. Similarly, detecting between-person mediation involving a log-residual variance requires N = 250, which is again lower than our sample size.
Data collection	The study was conducted from November 2023 to January 2024 and comprised a baseline survey, a 35-day experience sampling period, and a follow-up survey, respectively conducted before, during, and after the 2023 Christmas festive season. In November

2023, 380 parents from the United Kingdom, each with at least one child under the age of 10, were recruited via the cloud-sourcing platform Prolific. They first completed a baseline survey two weeks before being invited to download the MindSampler app on November 30, 2023. This application is designed for collecting intensive longitudinal data and integrates Qualtrics surveys. Participants received three randomly timed daily prompts between 8:00 and 20:00, from November 30, 2023 (Day 1) to January 3, 2024 (Day 35). Of those invited, 315 participants completed at least 30 daily experience sampling surveys throughout the study. On January 15, 2024, 307 of these participants also completed the follow-up survey. Participants received a total compensation of £30, distributed as follows: £4 for completing the baseline survey, £1 for installing the MindSampler app, £10 as a flat participation fee for responding to at least 30 notifications during the 35-day experience sampling period, and £5 for completing the follow-up survey. Additionally, participants who completed all three parts of the study received a £10 bonus.

Timing	from November 2023 to January 2024 and comprised a baseline survey, a 35-day experience sampling period (Day 1: November 30, 2023; Day 35: January 3, 2024), and a follow-up survey, respectively conducted before, during, and after the 2023 Christmas festive season
Data exclusions	Participants who had completely missing data on the variables of interest at baseline, during the experience sampling period, or at follow-up, as well as those who reported having no children residing in the household, were excluded from the final dataset.
Non-participation	83 participants dropped out (mostly discontinued during the ESM period; participants were not required to indicate the reason)
Randomization	N.A.

Reporting for specific materials, systems and methods

We require information from authors about some types of materials, experimental systems and methods used in many studies. Here, indicate whether each material, system or method listed is relevant to your study. If you are not sure if a list item applies to your research, read the appropriate section before selecting a response.

Materials & experimental systems

n/a	Involved in the study
☒	☐ Antibodies
☒	☐ Eukaryotic cell lines
☒	☐ Palaeontology and archaeology
☒	☐ Animals and other organisms
☒	☐ Clinical data
☒	☐ Dual use research of concern
☒	☐ Plants

Methods

n/a	Involved in the study
☒	☐ ChIP-seq
☒	☐ Flow cytometry
☒	☐ MRI-based neuroimaging

Plants

Seed stocks	N.A.
Novel plant genotypes	N.A.
Authentication	N.A.
